# An exploratory analysis of the associations of human milk oligosaccharides and N-/O-glycans with infectious and allergic outcomes in the first year of life: the Japanese Human Milk Study Cohort

**DOI:** 10.1007/s00394-026-04074-9

**Published:** 2026-08-07

**Authors:** Yuta Tsujimori, Toshiyuki Yamaguchi, Yasuhiro Toba, Hirofumi Fukudome, Fumihiko Sakai, Hiroshi M. Ueno, Satoshi Higurashi

**Affiliations:** 1 Department of Research and Development, Bean Stalk Snow Co., Ltd., Kawagoe, Japan; 2 Milk Science Research Institute, MEGMILK SNOW BRAND Co., Ltd., Kawagoe, Japan

**Keywords:** Human milk oligosaccharides, N-/O-glycans, Infant allergies, Infant infections

## Abstract

**Purpose:**

This exploratory study examined the associations of human milk oligosaccharides (HMOs) and N-/O-glycans in human milk with infectious and allergic outcomes during infancy.

**Methods:**

This prospective cohort study included 200 Japanese mother–infant dyads selected from the Japanese Human Milk Study Cohort. Human milk samples were collected at 1–2 months postpartum, and 11 HMOs and 28 N-/O-glycans were measured. HMOs were measured as absolute concentrations, whereas N-/O-glycans were expressed as relative abundances based on normalized LC–MS signal intensity. Infant histories of infections and allergies during the first year of life were assessed using self-administered questionnaires. Associations between milk glycan components and infant health outcomes were evaluated using Firth’s penalized likelihood logistic regression. To aid interpretation of multiple glycan-outcome comparisons, q values were calculated within each outcome at each step of the hierarchical analysis using the Benjamini–Hochberg procedure for controlling the false discovery rate.

**Results:**

In Firth logistic regression analyses, several milk glycans showed nominal associations with infectious or allergic outcomes. Total sialic acid, disialylated N-glycans (N51 and N57), and 6′-sialyllactose showed nominal associations with lower odds of bronchitis, whereas total HMO showed a nominal association with higher odds of otitis media. However, no individual HMO showed a nominal association with this outcome. For food allergy, 3-fucosyllactose, lacto-*N*-fucopentaose II, and fucosyl-sialyl O-glycan (O24) showed nominal associations with lower odds, whereas 2′-fucosyllactose showed a nominal association with higher odds in the overall analysis. However, all infants who developed food allergy were born to secretor mothers, and several food allergy findings were attenuated in the analysis restricted to secretor mothers. For atopic dermatitis, high-mannose–type N-glycans, particularly N12 and N33, showed nominal associations with higher odds. No glycan-outcome association had a q value below 0.05 within each outcome and step of the hierarchical analysis.

**Conclusion:**

Several human milk glycans showed nominal associations with infectious or allergic outcomes during infancy. However, none had a q value below 0.05 after false discovery rate correction. These findings should be interpreted as exploratory and hypothesis-generating and require validation in larger independent cohorts.

**Supplementary Information:**

The online version contains supplementary material available at https://doi.org/10.1007/s00394-026-04074-9.

## Introduction

Pediatric infectious and allergic diseases have increased globally in recent years, and their prevention remains a major public health challenge. These conditions can have long-term consequences for infant growth, development, and subsequent health, underscoring the need for a better understanding of early-life risk factors and preventive strategies. Breastfeeding is widely recognized as an important factor for infant health, and exclusive breastfeeding for the first 6 months is recommended by the World Health Organization [1]. Human milk contains a wide range of carbohydrate-based structures that contribute to infant health. These include N- and O-linked glycans (N-/O-glycans), which are primarily attached to milk proteins, and human milk oligosaccharides (HMOs), which occur as unconjugated glycans. Collectively, these glycans are thought to play important roles in shaping immune function and may contribute to protection against infectious and allergic diseases in infancy [2, 3].

HMOs represent the third largest solid fraction in human milk, following lactose and lipids. More than 200 individual HMOs have been identified, most of which are lactose-based structures modified with monosaccharides such as *N*-acetylglucosamine, galactose, fucose, and sialic acid (SA) [4]. Approximately 70% of human milk proteins are glycosylated, primarily via N*-* and O*-*linkages. N‑glycosylation occurs at asparagine residues, while O‑glycosylation takes place at serine or threonine residues. Based on monosaccharide composition and branching patterns, N*-*glycans are classified into high-mannose, complex, and hybrid types, while O-glycans are categorized into eight core structures. Moreover, the non-reducing termini of N-/O-glycans are frequently fucosylated or sialylated, contributing to structural diversity [5, 6]. The HMO profile varies markedly among individuals and is strongly influenced by maternal genetic background [7, 8]. Our previous studies have found that O‑glycan composition is influenced by maternal genetic factors [9]. One of the key genetic determinants of milk glycan composition is the secretor (*Se*) gene, which encodes fucosyltransferase 2 (FUT2). Mothers with an active *Se* locus are classified as secretors, and their milk is enriched with α1–2 fucosylated HMOs, such as 2′-fucosyllactose (2′-FL) and lacto-*N*-fucopentaose I (LNFP-I). In contrast, milk from non-secretor mothers, who lack functional FUT2 activity, contains little or no α1–2 fucosylated HMOs [7, 8]. Beyond HMOs, the maternal FUT2 status may influence the repertoire and abundance of O-glycans in human milk, potentially altering terminal fucosylation and sialylation patterns [9].

Previous studies have reported associations between milk glycan profile and infant health outcomes. Specific HMOs have been linked to either reduced or increased risk of allergic diseases, although the implicated glycans differ across conditions. For instance, cesarean-delivered infants at high genetic risk for allergy have a lower risk of immunoglobulin E (IgE)-associated eczema by 2 years of age when their mothers’ milk contains FUT2-dependent oligosaccharides [10]. Low concentrations of lacto-*N*-fucopentaose II (LNFP-II) have been linked to a higher risk of cow’s milk allergy (CMA) at 6 months, and differences in maternal secretor status have been observed between infants with delayed-onset and IgE-mediated CMA [11]. These findings suggest that specific glycan structures may influence allergic outcomes, although the direction and magnitude of associations appear to vary by disease type.

In contrast, the relationship between milk glycans and infant respiratory infections, such as bronchitis, has been less extensively studied. Some HMOs have been associated with infection prevalence; however, both positive and inverse associations have been reported, resulting in inconsistent findings across studies [12–14]. Overall, current evidence suggests that milk glycans may influence infant health, whereas data remain limited and inconclusive. Moreover, most prior studies have focused primarily on HMOs, with less attention given to N-/O-glycans. To address these gaps, we conducted an exploratory analysis to examine whether multiple glycan components in human milk—including HMOs as well as N-/O-glycans—are associated with infectious and allergic outcomes during infancy. Using data from 200 mother–infant dyads enrolled in a prospective Japanese cohort [15, 16], we quantified 11 HMOs and 28 N-/O-glycans in human milk samples collected 1–2 months postpartum. Infant histories of infectious and allergic diseases during the first year of life were assessed using mother-reported questionnaires, and associations between human milk glycan components and health outcomes were evaluated using Firth logistic regression models. This exploratory analysis was intended to identify hypothesis-generating patterns and directions of association between milk glycans and infant outcomes, rather than to draw definitive conclusions.

## Materials and methods

### Participants

The 200 mother–infant dyads included in this analysis were selected from the Japanese Human Milk Study, a prospective longitudinal cohort of breastfeeding women and their infants in Japan. The study was conducted in accordance with the Declaration of Helsinki, approved by the institutional review board of Fukuda Clinic (approval No. 20140621-03), and registered with the University Hospital Medical Information Network Clinical Trials Registry (UMIN ID: 000015494). Written informed consent was obtained from all participants prior to enrollment.

Details of the Japanese Human Milk Study have been reported previously [15, 16]. Briefly, between October 2014 and May 2019, a total of 1,210 breastfeeding mother–infant dyads were recruited from 73 medical institutions, including 16 hospitals and obstetric clinics, across Japan. Eligible participants were healthy women who were able to provide human milk without difficulty. Before enrollment, all mothers received a detailed explanation of the study procedures from trained staff and provided written informed consent. Of the 1,210 dyads initially recruited, 83 were excluded because of incomplete or invalid responses, and 5 were excluded because either the woman or her partner was not Japanese. The remaining 1,122 dyads constituted the eligible study population. Among these, 930 mothers were able to provide sufficient human milk samples (> 50 mL) when their infants were 1–2 months old by June 2020. For this study, 200 dyads were randomly selected from these 930 participants using computer-generated random numbers (Online Resource 1). No additional inclusion or exclusion criteria were applied after this random selection. Participants with missing data for a given outcome were excluded only from the corresponding outcome-specific analysis.

### Mother–infant dyad background information

Background information on mothers and infants was collected using self-administered questionnaires administered every 2 months, for up to six time points during the first postpartum year. Collected variables included sociodemographic characteristics (maternal age, education level, and household income), physical characteristics (prepregnancy body mass index [BMI]), smoking status, infant feeding method, mode of delivery, delivery experience, infant sex and birth weight, and parental history of allergic disease. Each questionnaire also assessed whether the infant had received a physician diagnosis of any infectious or allergic condition since the previous survey. Reported infections included gastroenteritis, bronchitis, otitis media, respiratory syncytial virus (RSV) infection, hand, foot, and mouth disease (HFMD), and exanthem subitum. Allergic outcomes included food allergy and atopic dermatitis. For each condition, a history was defined as present if the caregiver reported a physician diagnosis at least once during the first year of life. Participants who withdrew from follow-up without reporting any of these outcomes before withdrawal were coded as missing for the corresponding variables.

### Human milk samples

Detailed procedures for human milk collection in the Japanese Human Milk Study have been described previously [15]. In the present analysis, human milk samples collected from the 200 selected participants at 1–2 months postpartum were used. Milk samples were collected over a 7-day period, with milk from at least 3 collection days pooled for each participant. The pooled samples were aliquoted into 1-mL portions and stored at − 80 °C until analysis.

### Glycan analysis

Total SA concentrations in human milk were quantified using high-performance anion-exchange chromatography with pulsed amperometric detection (HPAEC-PAD). The analytical procedure is described below. Briefly, 800 µL of distilled water and 100 µL of 1 mol/L H_2_SO_4_ were added to 100 µL of human milk, and the mixture was incubated at 80 °C for 45 min. After hydrolysis, 1 mL of 0.1 mol/L NaOH was added to neutralize the sample. The mixture was then centrifuged at 15,000 × *g* for 10 min using a 10,000-molecular- weight- cutoff ultrafiltration membrane to remove proteins. The resulting hydrolysates were applied to a Dionex CarboPac PA1 column (4 mm internal diameter × 250 mm; 10 µm particle size; Thermo Fisher Scientific, Waltham, MA, USA). The mobile phase consisted of water (solvent A), 200 mM NaOH (solvent B), and 600 mM sodium acetate in 100 mM NaOH (solvent C), with gradient elution as follows: 0–7 min, 45% B and 10% C; 7–10 min, 45–37.5% B and 10–25% C; 10–20 min, 37.5% B and 25% C; 20–20.1 min, 37.5–45% B and 25–10% C; and 20.1–25 min, 45% B and 10% C, at a flow rate of 1 mL/min. Chromatograms were analyzed using Chromeleon software version 6.8 (Thermo Fisher Scientific).

**Fig. 1:**
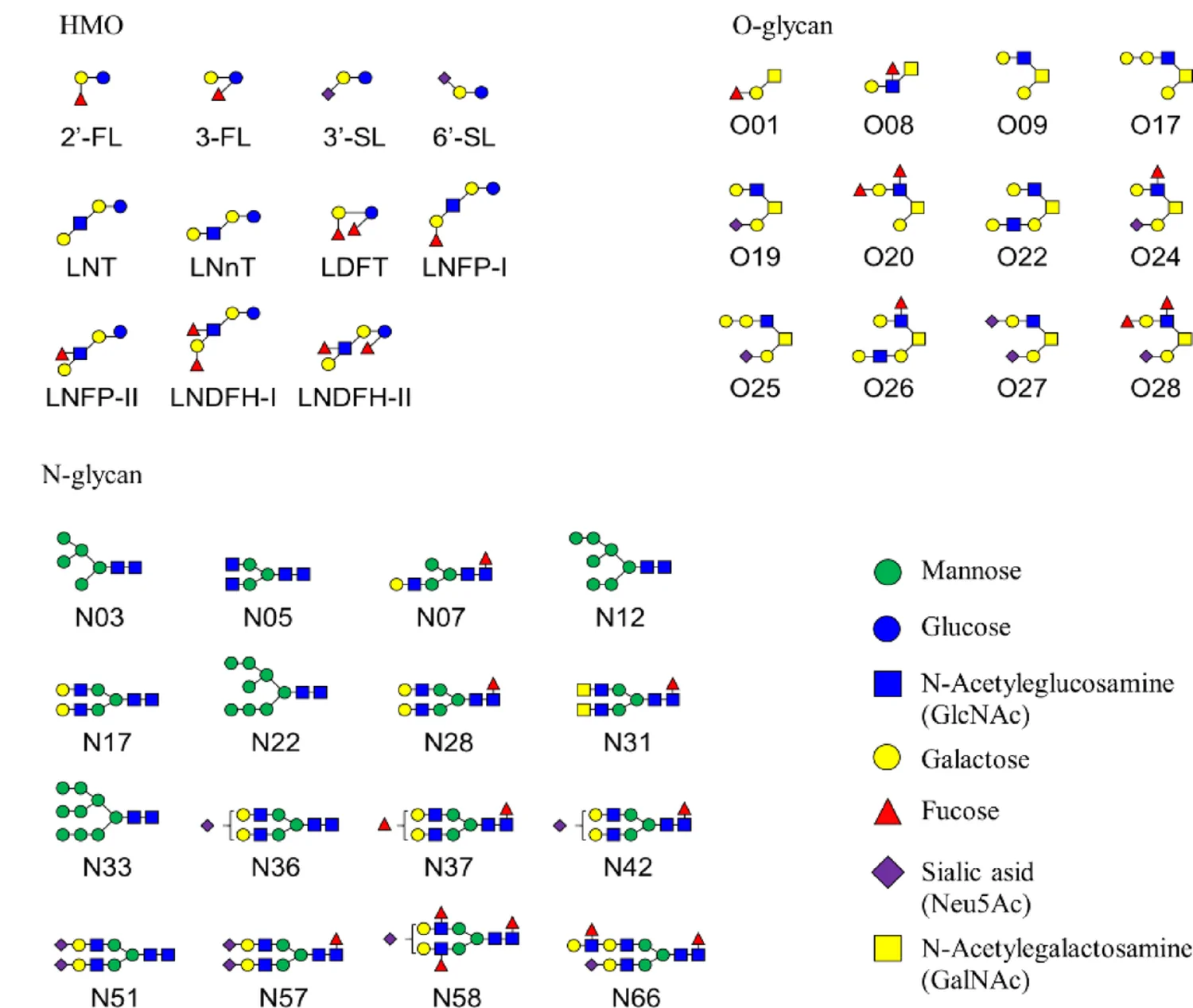
Structures of human milk oligosaccharides and N-/O*-*glycans. Structures of 16 N-glycans and 12 O-glycans quantified on a relative basis were assigned by LC–MS/MS analysis of human milk samples. The concentrations of 11 HMOs were quantified absolutely using HPAEC-PAD in human milk. 2′-FL, 2′-fucosyllactose; 3-FL, 3-fucosyllactose; 3′-SL, 3′-sialyllactose; 6′-SL, 6′-sialyllactose; LNT, lacto-*N*-tetrose; LNnT, lacto-*N*-neotetraose; LDFT, lactodifucotetraose; LNFP-I, lacto-*N*-fucopentaose I; LNFP-II, lacto-*N*-fucopentaose II; LNDFH-I, lacto-*N*-difucohexaose I; LNDFH-II, lacto-*N*-difucohexaose II; HMOs, human milk oligosaccharides; LC–MS/MS, liquid chromatography–tandem mass spectrometry; HPAEC-PAD, high-performance anion-exchange chromatography with pulsed amperometric detection

Analytical methods and results for other individual milk glycans have been reported previously [9]. Briefly, absolute concentrations of 11 HMOs were quantified using HPAEC-PAD whereas relative abundances of 28 N-/O-glycans were determined using label-free liquid chromatography–mass spectrometry (LC–MS) based on normalized signal intensity (Fig. 1).

### Statistical analyses

Maternal and infant characteristics according to the histories of infections and allergic diseases during the first year of life were summarized as medians and interquartile ranges (IQRs; Q1–Q3) for continuous variables and as numbers and percentages for categorical variables. Categorical variables were compared using Pearson’s chi-square test or Fisher’s exact test, as appropriate, and continuous variables were compared using the Wilcoxon rank-sum test. To evaluate potential selection bias arising from the multistage selection process, we conducted two assessments. First, the 930 dyads whose mothers provided sufficient milk samples were compared with the overall eligible cohort of 1,122 dyads. Second, among the 930 dyads, baseline characteristics were compared between the 200 dyads selected for this study and the remaining 730 eligible but non-selected dyads.

As an exploratory screening framework, we used a two-step hierarchical analytic strategy based on a priori grouping of glycans with shared structural or functional characteristics. In total, eight glycan groups were defined, each with a prespecified group-level summary variable (Table 1). Sialylated glycans, including sialylated HMOs such as 3′-sialyllactose (3′-SL) and 6′-sialyllactose (6′-SL), as well as sialylated N-/O-glycans, were grouped as “Sialylated glycans.” For this group, total SA concentration measured by HPAEC-PAD was used as a broad group-level marker of milk sialylation because it may include SA derived from both quantified and unquantified sialylated compounds. Similarly, fucosylated HMOs were grouped as “Fucosylated HMOs,” and the summed concentrations of individual fucosylated HMOs were used as the corresponding group-level summary variable. Analogous group-level definitions were applied to the remaining glycan groups (Table 1). Glycans exhibiting significant differences according to maternal secretor status were categorized into two additional groups. Because these groups included a mixture of absolute HMO concentrations and relative N-/O-glycan abundance values, group-level summary variables were not defined. Instead, maternal secretor status, determined by the concentration of 2′-fucosyllactose (2′-FL) as previously described [9], was used as a categorical variable in step 1 of the hierarchical analysis.

**Table 1:** Glycan groups, component glycans and group-level summary variables in 200 Japanese mother–infant dyads

Glycan group	Individual glycans	Median	IQR (Q1–Q3)	Min	Max
Sialylated glycans^1^ (mg/L)	3′-SL, 6′-SL, N36, N42, N51, N57, N58, N66, O19, O24, O25, O27, O28	372	292.3–488.4	179.6	977.1
Total HMO^2^ (mg/L)	2′-FL, 3-FL, 3′-SL, 6′-SL, LNT, LNnT, LDFT, LNFP-I, LNFP-II, LNDFH-I, LNDFH-II	4327	3620–5192	847.2	9016
Fucosylated HMO^2^ (mg/L)	2′-FL, 3-FL, LDFT, LNFP-I, LNFP-II, LNDFH-I, LNDFH-II	3459	2951–4144	329.8	6511
High-mannose–type N-glycan^2^	N03, N12, N22, N33	0.673	0.424–1.775	0.109	36
Complex-type N-glycan^2^	N05, N07, N17, N28, N31, N36, N37, N42, N51, N57, N58, N66	6.3	4.55–9.54	2.02	45.5
Fucosylated N-glycan^2^	N07, N28, N31, N37, N42, N57, N58, N66	1.21	0.839–1.783	0.271	11.3
Total O-glycan^2^	O01, O08, O09, O17, O19, O20, O22, O24, O25, O26, O27, O28	109.2	66.9–176.1	30.4	1475
Fucosylated O-glycan^2^	O01, O08, O20, O24, O26, O28	6.78	4.26–10.7	1.13	45.6
Glycan abundant in secretor human milk^3^	2'-FL, LNnT, LDFT, LNFP-Ⅰ, LNDFH-Ⅰ, N12, N28, N42, N58, O01, O20, O28	–	–	–	–
Glycan abundant in non-secretor human milk^3^	3-FL, LNT, LNFP-II, LNDFH-II, N31, N37, O08, O09, O24, O26, O27	–	–	–	–

^1^The group-level summary variable for sialylated glycans was total sialic acid concentration in human milk (mg/L) measured by HPAEC-PAD. Total sialic acid concentration was used as a broad marker of milk sialylation and may include sialic acid from both quantified and unquantified sialylated compounds

^2^N-/O-glycans were expressed as relative abundances based on LC–MS peak areas normalized to maltohexaose as an internal standard. Group abundances for N-/O-glycans were calculated by summing normalized peak areas of individual glycans within each group. Total HMOs and fucosylated HMOs were calculated as absolute concentrations (mg/L) by summing individual HMO concentrations measured using HPAEC-PAD

^3^Glycans classified as abundant in secretor or non-secretor human milk showed significant differences according to maternal secretor status (Mann–Whitney U test; *p* < 0.05)

HPAEC-PAD, high-performance anion-exchange chromatography with pulsed amperometric detection; LC–MS, liquid chromatography–mass spectrometry; 2′-FL, 2′-fucosyllactose; 3-FL, 3-fucosyllactose; 3′-SL, 3′-sialyllactose; 6′-SL, 6′-sialyllactose; LNT, lacto-*N*-tetrose; LNnT, lacto-*N*-neotetraose; LDFT, lactodifucotetraose; LNFP-I, lacto-*N*-fucopentaose I; LNFP-II, lacto-*N*-fucopentaose II; LNDFH-I, lacto-*N*-difucohexaose I; LNDFH-II, lacto-*N*-difucohexaose II; IQR, interquartile range

Associations between histories of infections and allergic diseases during the first year of life and milk glycan variables were evaluated using Firth’s penalized likelihood logistic regression to reduce small-sample bias and address potential complete or quasi-complete separation due to sparse outcome events. Both crude and multivariable adjusted Firth logistic regression models were fitted; the latter are referred to as adjusted models throughout the manuscript. For infection outcomes, adjusted models included delivery experience, mode of delivery, infant sex, and birth weight as covariates [13]. For allergic outcomes, parental history of allergic disease was additionally included as a covariate [10, 17, 18]. Regression analyses were conducted using complete cases for the outcome and covariates included in each model. The primary interpretation was based on the adjusted Firth logistic regression models. Continuous individual glycan variables and group-level summary variables were standardized to a mean of 0 and a standard deviation of 1 using z-score transformation before regression analyses. Odds ratios were therefore estimated per one-standard-deviation increase in each glycan variable or group-level summary variable. HMOs were measured as absolute concentrations, whereas N-/O-glycans were expressed as relative abundances based on normalized LC–MS signal intensity. Accordingly, odds ratios for HMOs should be interpreted as associations per one-standard-deviation increase in absolute concentration, whereas odds ratios for N-/O-glycans should be interpreted as associations per one-standard-deviation increase in relative abundance or normalized signal intensity.

In step 1 of the hierarchical analysis, associations between each group-level summary variable and the outcome were evaluated using adjusted Firth logistic regression models. For the secretor-associated and non-secretor-associated glycan groups, maternal secretor status was evaluated as the corresponding categorical variable. Glycan groups or maternal secretor status showing nominal associations, defined as multiplicity-unadjusted *p* values < 0.05 in adjusted models, proceeded to step 2. In step 2, associations between individual glycans within those groups and the outcome were examined using the same adjusted Firth logistic regression models as in step 1. When maternal secretor status showed a nominal association with food allergy in step 1, individual glycans that differed significantly between secretor and non-secretor mothers were examined in relation to food allergy in step 2. To aid interpretation of multiple glycan-outcome comparisons, q values were calculated within each outcome at each step of the hierarchical analysis using the Benjamini–Hochberg procedure for controlling the false discovery rate. Associations with multiplicity-unadjusted *p* values < 0.05 were described as nominal associations.

Sensitivity analyses were conducted for individual glycan–outcome pairs that showed nominal associations in step 2 of the hierarchical analysis. First, to evaluate the influence of potential outliers, glycan variables were standardized using z-score transformation, and potential outliers were handled based on these standardized values. Two outlier-handling approaches were applied: exclusion of values beyond 3 standard deviations from the mean and 1% winsorization at the 1st and 99th percentiles. Associations were then re-estimated using Firth logistic regression models with the same covariate adjustments used in the primary analysis. Second, reduced-covariate models were fitted for the same glycan–outcome pairs. For each glycan–outcome pair, separate Firth logistic regression models were fitted using reduced covariate sets rather than the full set of covariates used in the primary adjusted model. These included models with one covariate at a time, as well as a model including both infant sex and mode of delivery. The single covariates examined were delivery experience, mode of delivery, infant sex, and birth weight for infectious outcomes; parental history of allergic disease was additionally examined for allergic outcomes. These reduced-covariate models were used as sensitivity analyses to evaluate whether the direction of association was consistent when model complexity was reduced under sparse-event conditions. Finally, for food allergy, a sensitivity analysis restricted to secretor mothers was conducted. Candidate glycans were selected from individual glycans that showed nominal associations with food allergy in step 2 of the hierarchical analysis. These glycan–food allergy associations were re-evaluated among secretor mothers using Firth logistic regression models with the same covariate adjustments as those used for food allergy in step 2. Statistical analyses were performed using SPSS Statistics version 27.0 (IBM Corp., Armonk, NY, USA) and R version 4.5.3 (R Foundation for Statistical Computing, Vienna, Austria).

## Results

### Participant characteristics

Baseline characteristics and infant outcomes of the 200 mother-infant dyads selected for this study and the remaining 730 eligible but non-selected dyads among the 930 dyads whose mothers provided sufficient milk samples are shown in Table 2. Among the infants included in this study, 60% (121/200) were male, and 7.1% (14/196) were delivered by cesarean section. The median birth weight was 3080 g, and 2.1% (4/190) of infants were born preterm (gestational age < 37 weeks). With respect to feeding practices, 86% (171/199) of infants were exclusively breastfed. Because not all participants completed every follow-up questionnaire, denominators varied across outcomes. During the first year of life, the reported frequencies of infectious and allergic outcomes were as follows: gastroenteritis, 19% (30/155); bronchitis, 12% (18/153); otitis media, 17% (26/155); RSV infection, 15% (23/157); HFMD, 16% (26/158); exanthem subitum, 26% (41/159); food allergy, 12% (18/150); and atopic dermatitis, 6.6% (10/152). Among mothers in this study, the median age at delivery was 32 years (IQR, 28–34 years), and 14% (28/196) were underweight before pregnancy (BMI < 18.5). Multiparous women accounted for 66% (130/198), and 68% (135/200) reported no history of smoking before pregnancy. Regarding socioeconomic characteristics, 33% (65/199) had completed a 4-year college education or higher, and 59% (115/194) reported a household income between 4 and 8 million yen. Overall, 80% (161/200) of mothers were classified as secretors. With respect to parental allergy history, 71% (139/195) of families reported that either the mother or her partner had a history of at least one allergic disease, including asthma, food allergy, atopic dermatitis, allergic rhinitis, allergic conjunctivitis, or urticaria. Assessment of infections and allergic diseases was based on six follow-up questionnaires administered over the first year of life; therefore, the denominators presented in Table 2 reflect the number of participants with available data for each outcome rather than only those who completed all follow-up surveys. When the 200 selected dyads were compared with the remaining 730 eligible but non-selected dyads, the two groups were generally comparable in maternal characteristics, birth characteristics, feeding method, parental allergic history, and reported infant outcomes. However, statistically significant differences were observed in infant sex and mode of delivery. No statistically significant differences were observed in the frequencies of any infectious or allergic outcomes examined between the selected and non-selected groups (Table 2).

**Table 2:** Comparison of baseline characteristics and reported infant outcomes between selected and non-selected dyads among the 930 dyads whose mothers provided sufficient milk samples^1^

	Non-selected dyads^2^ (*N* = 730)		Selected dyads (*N* = 200)		*p* value^3^
	*N*	*n* (%) or median (IQR)	*N*	*n* (%) or median (IQR)	
Infant characteristics					
Sex	730		200		0.029
Male		376 (52%)		121 (60%)	
Female		354 (48%)		79 (40%)	
Mode of delivery	729		196		0.044
Vaginal		637 (87%)		182 (93%)	
Cesarean section		92 (13%)		14 (7.1%)	
Birth weight (g)	720	3,056 (2,831, 3,304)	197	3,080 (2,872, 3,296)	0.523
Gestational age (weeks)	705		190		0.770
< 37		21 (3.0%)		4 (2.1%)	
37–41		681 (97%)		185 (97%)	
≥ 42		3 (0.4%)		1 (0.5%)	
Feeding status	728		199		0.530
Exclusive breastfeeding (≥ 90%)		640 (88%)		171 (86%)	
Non-exclusive breastfeeding (< 90%)		88 (12%)		28 (14%)	
Infectious outcomes					
Gastroenteritis	537	87 (16%)	155	30 (19%)	0.423
Bronchitis	532	52 (10%)	153	18 (12%)	0.572
Otitis media	536	92 (17%)	155	26 (17%)	> 0.999
RSV infection	543	82 (15%)	157	23 (15%)	0.990
HFMD	539	59 (11%)	158	26 (16%)	0.085
Exanthem subitum	545	130 (24%)	159	41 (26%)	0.693
Allergic outcomes					
Food allergy	535	68 (13%)	150	18 (12%)	0.926
Atopic dermatitis	534	30 (5.6%)	152	10 (6.6%)	0.523
Maternal characteristics					
Age at delivery	730	31 (28, 34)	200	32 (28, 34)	0.913
Prepregnancy BMI (kg/m^2^)	726		196		0.736
< 18.5		119 (16%)		28 (14%)	
18.5–24.9		554 (76%)		152 (78%)	
≥ 25		53 (7.3%)		16 (8.2%)	
Delivery experience	727		198		0.875
Primiparous		243 (33%)		68 (34%)	
Multiparous		484 (67%)		130 (66%)	
Smoking status	728		200		0.766
Never smoked		508 (70%)		135 (68%)	
Former smoker (quit before pregnancy)		206 (28%)		62 (31%)	
Smoked during pregnancy		14 (1.9%)		3 (1.5%)	
Education level	724		199		0.553
JHS/HS		160 (22%)		50 (25%)	
Some college/technical school		302 (42%)		84 (42%)	
4-year college or graduate degree		262 (36%)		65 (33%)	
Household income from all sources (million JPY/year)	703		194		0.335
< 4		184 (26%)		57 (29%)	
4 to < 8		412 (59%)		115 (59%)	
≥ 8		107 (15%)		22 (11%)	
Secretor status (secretor)^5^	–	–	200	161 (80%)	–
Parental history of allergic disease^4^	711	476 (67%)	195	139 (71%)	0.530

^1^Differences between the total *N* for a variable and the number of selected or non-selected dyads reflect missing data for that variable

^2^Non-selected dyads were the 730 mother–infant dyads whose mothers provided sufficient human milk samples but were not selected for this study

^3^Continuous variables were compared using the Wilcoxon rank-sum test; categorical variables were compared using Pearson’s chi-square test or Fisher’s exact test, as appropriate

^4^Parental history of allergic disease included asthma, food allergy, atopic dermatitis, allergic rhinitis, allergic conjunctivitis, and urticaria

^5^Secretor status was determined only for the selected dyads because glycan analyses were performed in these samples

RSV, respiratory syncytial virus; HFMD, hand, foot, and mouth disease; BMI, body mass index; JHS, junior high school; HS, high school; JPY, Japanese yen

Baseline characteristics and reported infant outcomes of the 930 dyads whose mothers provided sufficient milk samples are descriptively summarized alongside those of the overall eligible cohort of 1,122 dyads in Online Resource 2. Overall, the distributions appeared broadly similar across maternal characteristics, birth characteristics, feeding method, parental allergic history, and reported infant outcomes.

### Glycan group profiles

The glycan group-level summary variables in human milk from mothers in the 200 selected dyads are summarized in Table 1. HMO variables represent absolute concentrations, whereas N-/O-glycan variables represent relative abundance based on normalized LC–MS signal intensity. Median values (minimum–maximum) were as follows: sialylated glycans, 372.0 mg/L (179.6–977.1); total HMOs, 4327 mg/L (847.2–9016); fucosylated HMOs, 3459 mg/L (329.8–6511); high-mannose–type N-glycans, 0.673 (0.109–36); complex-type N-glycans, 6.30 (2.02–45.5); fucosylated N-glycans, 1.21 (0.271–11.3); total O-glycans, 109.2 (30.4–1475); and fucosylated O-glycans, 6.78 (1.13–45.6).

### Associations between glycan groups and infectious outcomes

**Fig. 2:**
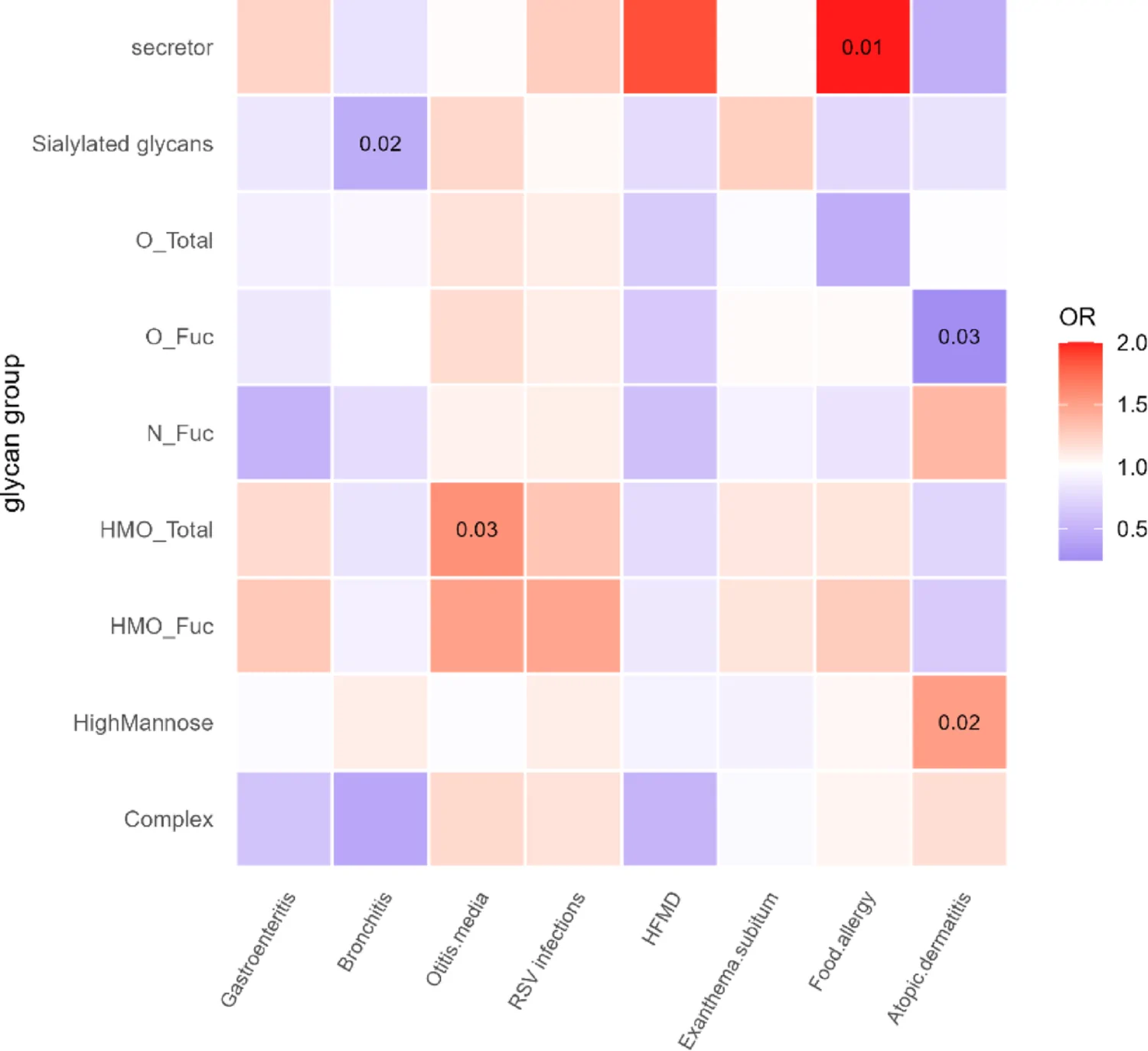
Associations between glycan groups in human milk at 1–2 months postpartum and infectious and allergic outcomes during the first year of life. Associations were evaluated using adjusted Firth logistic regression models in the selected Japanese mother–infant dyads with available data for each outcome. Explanatory variables included one glycan group-level summary variable or maternal secretor status. Glycan groups were categorized by structural characteristics, including human milk oligosaccharides (HMOs) and N-/O-glycans. For infectious outcomes, models were adjusted for delivery experience, mode of delivery, infant sex, and birth weight; for allergic outcomes, parental history of allergic disease was additionally included as a covariate. Cell colors represent odds ratios (ORs), with red indicating OR > 1 and blue indicating OR < 1; darker shading reflects greater deviation from the null value (OR = 1). Color intensity alone does not indicate statistical significance. Only associations with multiplicity-unadjusted *p* values < 0.05 are annotated within the cells. The q values are provided in Online Resource 4. Secretor, maternal secretor status; HMO_total, total human milk oligosaccharides; HMO_Fuc, fucosylated HMO; HighMannose, high-mannose–type N-glycans; Complex, complex-type N-glycans; N_Fuc, fucosylated N-glycans; O_Total, total O-glycans; O_Fuc, fucosylated O-glycans; OR, odds ratio

Associations between glycan group-level summary variables in human milk collected at 1–2 months postpartum and infectious outcomes during the first year of life were evaluated using Firth logistic regression. Results from crude and adjusted models are shown in Online Resource 3 and Fig. 2. Consistent with the prespecified analytic approach, interpretation was based primarily on adjusted models.

**Fig. 3:**
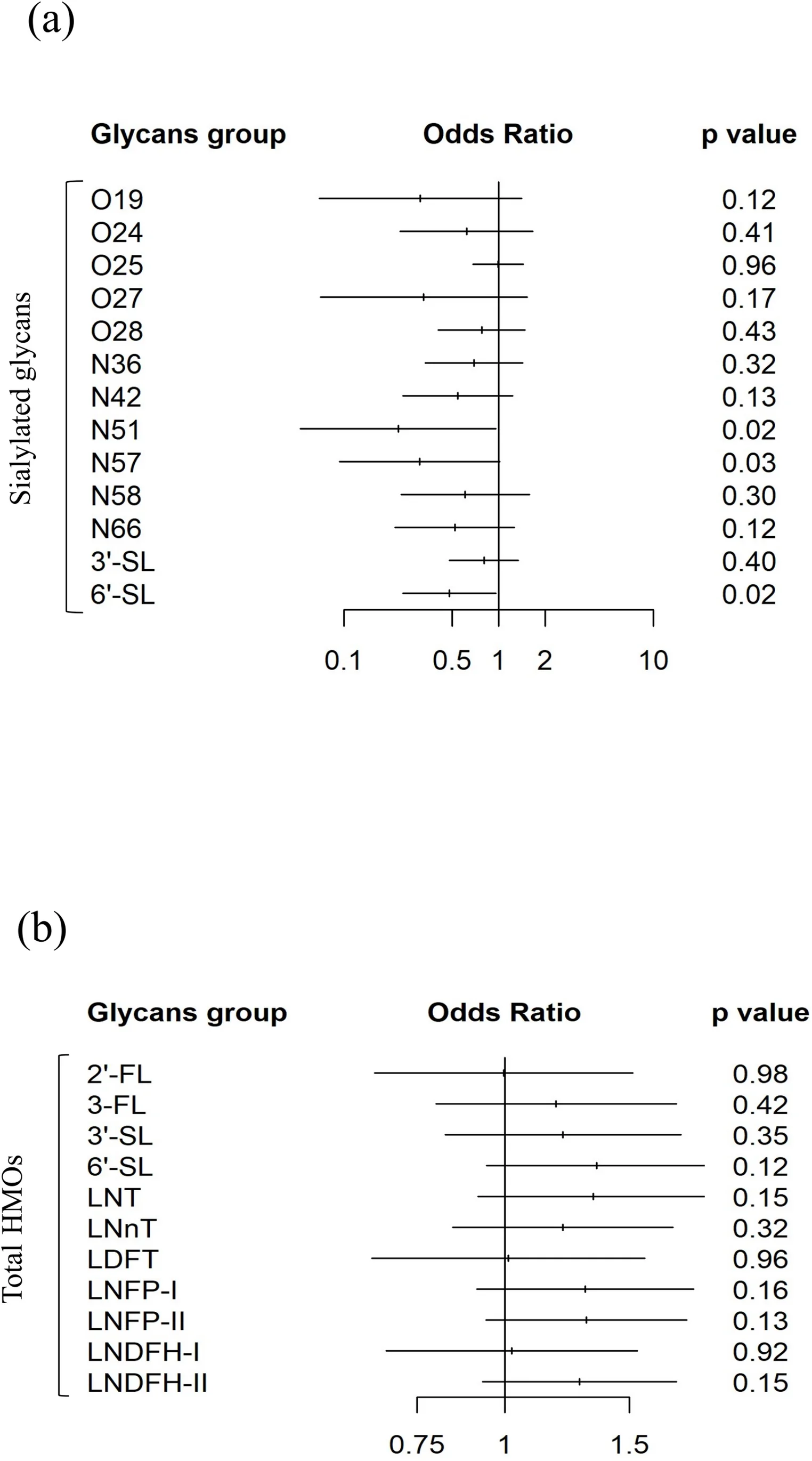
Odds ratios (ORs) and 95% confidence intervals (CIs) for bronchitis and otitis media during the first year of life. Forest plots show ORs and 95% CIs estimated using adjusted Firth logistic regression models that included delivery experience, mode of delivery, infant sex, and birth weight as covariates. In panel (**a**), individual sialylated glycans were included as explanatory variables to evaluate associations with bronchitis. In panel (**b**), individual HMOs were included as explanatory variables to evaluate associations with otitis media. *Abbreviations*: HMO, human milk oligosaccharides; OR, odds ratio; CI, confidence interval; 2′-FL, 2′-fucosyllactose; 3-FL, 3-fucosyllactose; 3′-SL, 3′-sialyllactose; 6′-SL, 6′-sialyllactose; LNT, lacto-*N*-tetrose; LNnT, lacto-*N*-neotetraose; LDFT, lactodifucotetraose; LNFP-I, lacto-*N*-fucopentaose I; LNFP-II, lacto-*N*-fucopentaose II; LNDFH-I, lacto-*N*-difucohexaose I; LNDFH-II, lacto-*N*-difucohexaose II

For bronchitis, total SA concentration was nominally associated with lower odds of bronchitis (odds ratio [OR] = 0.46, 95% confidence interval [CI] = 0.20–0.90, *p* = 0.021) (Fig. 2, Online Resource 4). In the subsequent individual-glycan analysis, 6′-SL (OR = 0.48, 95% CI = 0.20–0.92, *p* = 0.024) and disialylated N-glycans N51 (OR = 0.22, 95% CI = 0.03–0.88, *p* = 0.022) and N57 (OR = 0.31, 95% CI = 0.06–0.93, *p* = 0.031) were nominally associated with lower odds of bronchitis. Other sialylated glycans also tended to have ORs below 1, although their nominal p values were above 0.05 (Fig. 3, Online Resource 5). In sensitivity analyses, the nominal associations for 6′-SL, N51, and N57 were generally unchanged after outlier handling and in reduced-covariate Firth logistic regression models, with ORs remaining below 1. Detailed results are shown in Online Resources 6 and 7.

For otitis media, total HMO concentration showed a nominal positive association in the adjusted model (OR = 1.58, 95% CI = 1.05–2.42, *p* = 0.028), whereas no individual HMO showed an association (Fig. 3). No nominal associations were observed between any glycan group and other infectious outcomes, including gastroenteritis, RSV infection, HFMD, or exanthem subitum. Overall, several glycan variables showed nominal associations with infectious outcomes, but none had a q value below 0.05 within each outcome and step of the hierarchical analysis (Online Resources 4 and 5).

### Associations between glycan groups and allergic outcomes

Associations between glycan group-level summary variables in human milk collected at 1–2 months postpartum and allergic outcomes during the first year of life were evaluated using Firth logistic regression. Results from crude and adjusted models are shown in Online Resource 3 and Fig. 2. Consistent with the prespecified analytic approach, interpretation was based primarily on adjusted models.

**Fig. 4:**
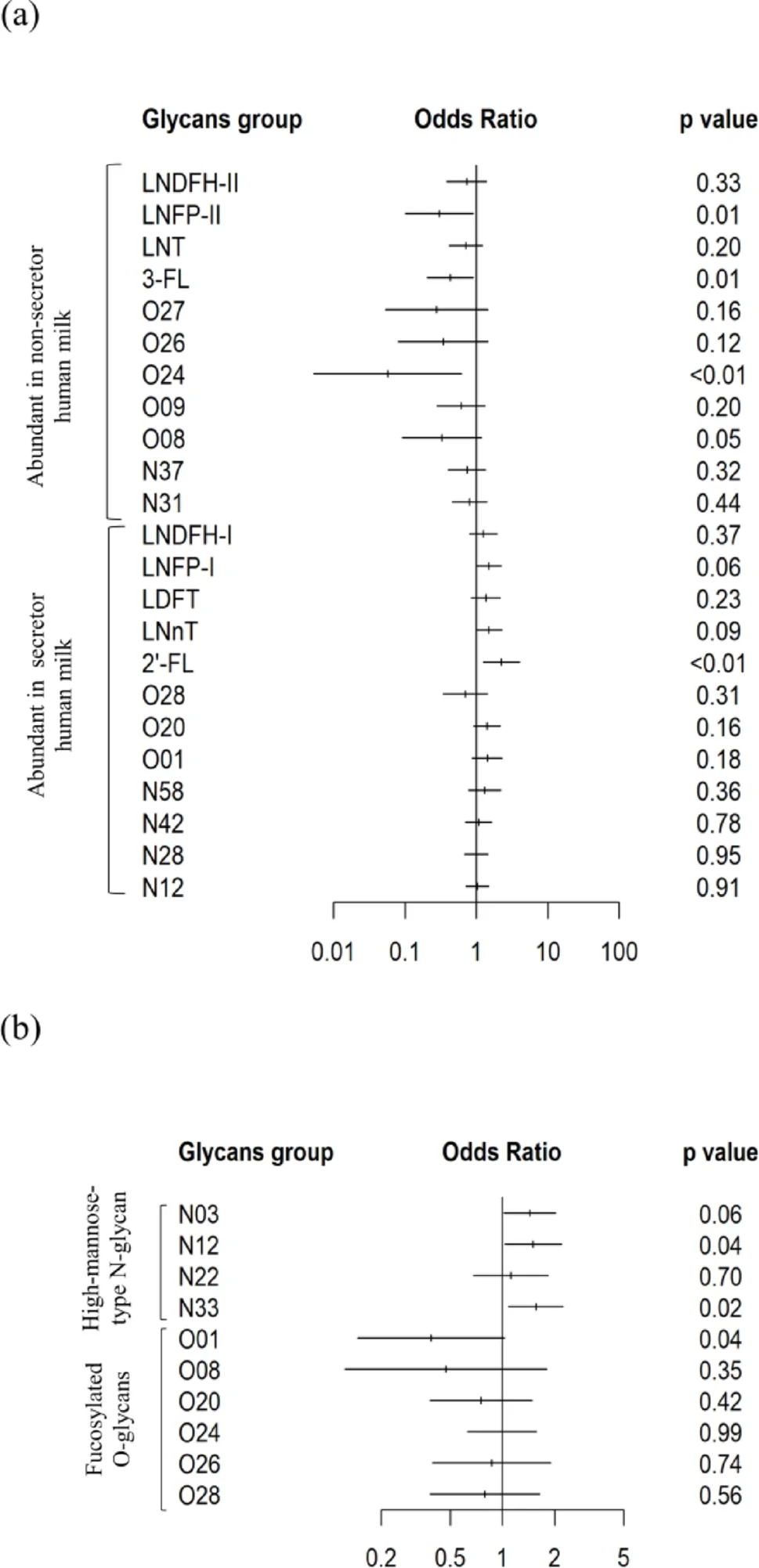
Odds ratios (ORs) and 95% confidence intervals (CIs) for food allergy and atopic dermatitis during the first year of life. Forest plots show ORs and 95% CIs estimated using adjusted Firth logistic regression models that included delivery experience, mode of delivery, infant sex, birth weight, and parental history of allergic disease as covariates. In panel (**a**), individual glycans that differed according to maternal secretor status (Mann–Whitney U test, *p* < 0.05) were included as explanatory variables to evaluate associations with food allergy. In panel (**b**), individual high-mannose–type N-glycans and fucosylated O-glycans were included as explanatory variables to evaluate associations with atopic dermatitis. Glycan variables were standardized before analysis. OR, odds ratio; CI, confidence interval; 2′-FL, 2′-fucosyllactose; 3-FL, 3-fucosyllactose; LNT, lacto-*N*-tetrose; LNnT, lacto-*N*-neotetraose; LDFT, lactodifucotetraose; LNFP-I, lacto-*N*-fucopentaose I; LNFP-II, lacto-*N*-fucopentaose II; LNDFH-I, lacto-*N*-difucohexaose I; LNDFH-II, lacto-*N*-difucohexaose II

For atopic dermatitis, the high-mannose–type N-glycan group showed a nominal positive association in the adjusted model (OR = 1.51, 95% CI = 1.06–2.29, *p* = 0.023). In the subsequent individual-glycan analysis, N12 (OR = 1.50, 95% CI = 1.02–2.36, *p* = 0.040) and N33 (OR = 1.56, 95% CI = 1.08–2.39, *p* = 0.019) showed nominal positive associations with atopic dermatitis (Fig. 4, Online Resource 5). In contrast, fucosylated O-glycans showed a nominal inverse association with atopic dermatitis (OR = 0.25, 95% CI = 0.04–0.90, *p* = 0.030). In the individual fucosylated O-glycan analysis, O01 showed a nominal inverse association with atopic dermatitis in the adjusted model (OR = 0.39, 95% CI = 0.09–0.98, *p* = 0.043) (Fig. 4, Online Resource 5). Sensitivity analyses using outlier handling and reduced-covariate models showed broadly similar directions for the atopic dermatitis findings, although the nominal associations were not consistently retained across all analyses. Detailed results are shown in Online Resources 6 and 7.

For food allergy, maternal secretor status showed a nominal association in the adjusted model, although the confidence interval was very wide (OR = 11.5, 95% CI = 1.46–1488, *p* = 0.014). In the subsequent individual-glycan analysis, 3-FL (OR = 0.43, 95% CI = 0.17–0.85, *p* = 0.012), LNFP-II (OR = 0.30, 95% CI = 0.07–0.83, *p* = 0.012), and O24 (OR = 0.06, 95% CI = 0.003–0.58, *p* = 0.008) showed nominal inverse associations with food allergy, whereas 2′-FL (OR = 2.22, 95% CI = 1.25–4.40, *p* = 0.006) showed a nominal positive association with food allergy(Fig. 4, Online Resource 5). However, none of these associations had q values below 0.05 (Online Resource 5). Because all food allergy cases occurred among infants born to secretor mothers (Online Resource 8), a sensitivity analysis restricted to secretor mothers was conducted. In the analysis, the nominal associations observed for 3-FL and LNFP-II in the overall analysis were attenuated and were no longer nominally associated with food allergy. The association for O24 was also attenuated but remained nominally associated with food allergy in the direction of lower odds (OR = 0.11, 95% CI = 0.01–0.93, *p* = 0.040) (Online Resource 9). In sensitivity analyses using outlier handling and in reduced-covariate models, the directions of the step 2 estimates were generally similar, although these results were interpreted together with the secretor-restricted sensitivity analysis because of the complete clustering of food allergy cases among infants born to secretor mothers (Online Resources 6 and 7). Overall, several glycan variables showed nominal directional associations with allergic outcomes, but none had q values below 0.05 within each allergic outcome (Online Resource 5).

## Discussion

This exploratory study evaluated associations between human milk glycans and infectious and allergic outcomes during the first year of life in Japanese mother–infant dyads. To our knowledge, this is one of the first studies to comprehensively evaluate the relationships between human milk glycans and infant health outcomes. Several milk glycans showed nominal associations with infant outcomes in Firth logistic regression analyses, with odds ratios indicating different directions depending on the glycan class and outcome. However, none of the associations had q values below 0.05 within each outcome and step of hierarchical analysis, and some findings were sensitive to maternal secretor status or outlier handling. Given the exploratory design of this study, these results should be viewed as hypothesis-generating rather than confirmatory.

For infectious outcomes, several sialylated glycans, including 6′-SL and disialylated N-glycans N51 and N57, showed nominal associations with lower odds of bronchitis. These bronchitis findings were generally consistent in sensitivity analyses. These findings are consistent with the hypothesis that certain sialylated human milk glycans may interact with pathogens or host mucosal surfaces. Glycans can function as soluble decoy receptors that may interfere with pathogen adhesion [2, 3]. Several respiratory pathogens recognize sialylated motifs, and α2,6–linked SAs, which are abundant on upper airway epithelial glycoconjugates, serve as attachment determinants for multiple viral strains [19–21]. Because 6′-SL contains an α2,6–linked SA, it could competitively inhibit pathogen binding. Although the specific SA linkages of N51 and N57 were not directly characterized in this study, α2,3-linked SA has not been reported in human milk N-glycan profiles [22], suggesting that α2,6–linked SA is a plausible structural feature of these disialylated N-glycans. The observation that disialylated, but not monosialylated, N-glycans showed inverse associations with bronchitis may reflect linkage- and topology-dependent avidity effects described in pathogen–glycan interactions [20]. These nominal associations may help generate hypotheses for future studies of respiratory infections and milk glycan structures.

For otitis media, higher total HMO concentration showed a nominal positive association, whereas no individual HMO showed a nominal association with this outcome, consistent with a previous report [13]. This group-level finding should be interpreted cautiously. Breastfeeding is known to reduce the risk of otitismedia and RSV infection [23, 24]. The finding does not detract from the well-established benefits of breastfeeding, which arise from a complex interplay of immunoglobulins, lactoferrin, lysozyme, cytokines, and glycans [23–25]. Prior studies have suggested that HMO–pathogen interactions may be context-dependent, with the possibility that higher HMO concentrations could stabilize certain viral structures or be exploited by pathogens to facilitate cellular entry [26]. Future studies that jointly evaluate HMOs, other bioactive milk components, pathogen-specific outcomes, and host factors may help clarify this relationship.

For atopic dermatitis, high-mannose–type N-glycans showed nominal positive associations, particularly N12 and N33. Fucosylated O-glycansshowed a nominal inverse association with atopic dermatitis at the group level, and O01 showed a nominal association in the same direction in the individual fucosylated O-glycan analysis. Sensitivity analyses showed broadly similar directions for these findings, but the nominal associations were not consistently retained across all analyses. These observations may generate hypotheses regarding human milk glycan structural diversity. One possible interpretation is that high-mannose-type N-glycan findings may reflect differences in glycan processing, potentially involving Golgi processing of high-mannose precursors into more complex or terminally modified glycans [27]. In contrast, the biological interpretation of the fucosylated O-glycan findings remains unclear based on the present data. However, glycan biosynthetic pathways, glycoprotein carriers, and immune mechanisms were not directly evaluated in this study. Therefore, this interpretation remains speculative and should be regarded as hypothesis-generating.

All infants who developed food allergy were born to secretor mothers. In step 1 of the hierarchical analysis, maternal secretor status showed a nominal association with higher odds of food allergy. However, the confidence interval was very wide (OR = 11.5, 95% CI = 1.46–1488), reflecting the sparse data structure and the absence of cases among infants born to non-secretor mothers. Accordingly, individual glycans that differed between secretor and non-secretor mothers were examined in relation to food allergy.

In the analysis of individual glycans, 3-FL, LNFP-II, and O24, which were previously shown to be more abundant in non-secretor milk in our glycan profiling analysis [9], showed nominal associations with lower odds of food allergy. In contrast, higher 2′-FL showed a nominal association with higher odds of food allergy. These results were generally consistent in sensitivity analyses using outlier handling and reduced-covariate models. Maternal secretor status was defined by 2′-FL concentration, and 2′-FL and 3-FL were inversely correlated in this cohort (Spearman’s rank correlation, *ρ* =  − 0.69). Thus, the 2′-FL finding may partly reflect maternal secretor phenotype or lower exposure to non-secretor-associated glycans rather than an effect of 2′-FL itself. In the sensitivity analysis restricted to secretor mothers, the nominal associations observed for 3-FL, LNFP-II, and 2’-FL in the overall analysis were attenuated and no longer reached nominal association. The association for O24 was also attenuated compared with the overall analysis, but it remained nominally associated with lower odds of food allergy. This pattern may suggest that the food allergy findings cannot be attributed solely to a simple contrast between maternal secretor phenotype and non-secretor phonotypes, although the small number of events precludes firm interpretation.

The results of LNFP-II and 2′-FL in the overall analysis are partly consistent with previous studies reporting associations between LNFP-II and allergic outcomes, as well as variable associations for 2′-FL across cohorts [18, 28]. Several biological mechanisms may hypothetically link human milk glycans to infant allergic outcomes. HMOs have been proposed to influence allergic disease development through multiple pathways, including modulation of gut microbiota, intestinal barrier function, immune responses, and lectin-mediated interactions [29]. The glycans 3-FL, LNFP-II, and O24, for which a nominal association was identified in this study, share structural features resembling Lewis X–like motifs, characterized by branched terminal galactose and fucose residues. Dendritic cell (DC)-specific intercellular adhesion molecule-3–grabbing nonintegrin (DC-SIGN), a C-type lectin expressed on DCs, recognizes Lewis X structures and plays a role in antigen uptake and presentation [30]. These glycans could hypothetically interfere with allergen binding to C-type lectins such as DC-SIGN in the gastrointestinal tract, thereby modulating immune sensitization. In addition, 3-FL has been reported to enhance mucus production and intestinal barrier function, potentially through mechanisms involving the *MUC2* gene [31]. However, given the complete clustering of food allergy cases among infants born to secretor mothers, the very wide confidence interval for maternal secretor status, the attenuation of several HMO-related findings in the secretor-restricted analysis, and the lack of q values below 0.05, these mechanistic interpretations remain speculative and require further validation.

One of the notable strengths of this study is the comprehensive analysis of both HMOs and N-/O-glycans in human milk in relation to infectious and allergic outcomes. Moreover, the focus on a Japanese cohort provides valuable insights from a high-income Asian population with distinct genetic, environmental, and dietary characteristics compared with Western and other Asian cohorts. Such population-specific factors may influence milk glycan composition and infant health outcomes. Finally, the prospective design and repeated follow-up with detailed epidemiological data on multiple infections and allergic diseases enabled longitudinal assessment of associations between early-life milk glycan profiles and health outcomes during infancy.

This study has several important limitations. First, the sample size was limited, and the number of outcome events was small, particularly for allergic outcomes. Although Firth logistic regression was used to reduce small-sample bias and address potential separation, it does not eliminate the limited-information problem arising from sparse outcome events. Some estimates had wide confidence intervals, reflecting model instability and limited precision. This issue was particularly relevant for food allergy, for which all cases occurred among infants born to secretor mothers and the confidence interval for maternal secretor status was extremely wide. Although a sensitivity analysis restricted to secretor mothers was conducted, the number of events remained small, limiting our ability to distinguish glycan-specific associations from the influence of maternal secretor phenotype. For atopic dermatitis, findings involving high-mannose-type N-glycans were partly sensitive to outlier handling, particularly for N12. In addition, multiple glycan-outcome comparisons were examined, and none had q values below 0.05 within each outcome and step of the hierarchical analysis. Second, the interpretation of odds ratios is limited by differences in glycan measurement scales. HMOs were measured as absolute concentrations, whereas N-/O-glycans were expressed as relative abundances based on normalized LC–MS signal intensity. Therefore, odds ratios for HMOs and N-/O-glycans should not be interpreted as directly comparable quantitative estimates. Third, bioactive milk proteins such as immunoglobulins and lactoferrin were not quantified, and the specific glycoproteins carrying the analyzed N-/O-glycans were not identified. Infant milk intake volume was also not collected, which may affect the interpretation of glycan exposure. In addition, infectious and allergic outcomes were based on caregiver-reported physician diagnoses and were not confirmed by medical record review, which may have introduced outcome misclassification. Moreover, milk samples were collected at a single time point at 1–2 months postpartum. Because milk glycan composition changes during lactation, this single measurement may not fully represent glycan exposure throughout the first year of life and may have introduced exposure misclassification. Reverse causation and temporality should also be interpreted cautiously for all outcomes, as some infants may have developed symptoms or illnesses before or around the time of milk sampling, which may have influenced feeding practices or milk intake. This concern may be particularly relevant for atopic dermatitis, because skin symptoms can develop early in infancy and may have preceded or coincided with milk sampling. Finally, residual confounding and selection bias cannot be excluded. Information on daycare or nursery attendance was not available in the analytic dataset, and residual confounding by unmeasured postnatal environmental factors may remain, particularly for infectious outcomes. Although the 930 dyads whose mothers provided sufficient milk samples were broadly similar to the eligible cohort and the 200 selected dyads were generally comparable to the 730 non-selected dyads, differences in infant sex and mode of delivery were observed between the selected and non-selected groups. Therefore, residual selection bias and limited generalizability should be considered. Given the exploratory nature of this study and the limitations noted above, the findings should be interpreted cautiously and regarded as hypothesis-generating.

## Supplementary Information

Below is the link to the electronic supplementary material.

Supplementary Material 1

## Data Availability

The datasets used and/or analyzed during the current study are available from the corresponding author on reasonable request.
