# Supplementary material for: An exploratory analysis of the associations of human milk oligosaccharides and N-/O-glycans with infectious and allergic outcomes in the first year of life: the Japanese Human Milk Study Cohort
Source: Eur J Nutr. 2026 Aug 7;65(6):233. doi: 10.1007/s00394-026-04074-9 (PMC13451480; doi:10.1007/s00394-026-04074-9)
Supplement: Supplementary file 1 [file 394_2026_4074_MOESM1_ESM.docx]

**Supplementary Information (SI)**

***European Journal of Nutrition***

**An exploratory analysis of the associations of human milk oligosaccharides and N-/O-glycans with infectious and allergic outcomes in the first year of life: The Japanese Human Milk Study Cohort**

**Author details**

Yuta Tsujimori^1,2^, Toshiyuki Yamaguchi^1,2^, Yasuhiro Toba^1,2^, Hirofumi Fukudome^2^, Fumihiko Sakai^2^, Hiroshi M. Ueno^1,2^, Satoshi Higurashi^1^

1 Department of Research and Development, Bean Stalk Snow Co., Ltd., Kawagoe, Japan

2 Milk Science Research Institute, MEGMILK SNOW BRAND Co., Ltd., Kawagoe, Japan

E-mail addresses

Yuta Tsujimori, yuta-tsujimori@meg-snow.com

Toshiyuki Yamaguchi, toshiyuki-yamaguchi@beanstalksnow.co.jp

Satoshi Higurashi, s-higurashi@beanstalksnow.co.jp

Corresponding Author:

Satoshi Higurashi

1-1-2 Minamidai, Kawagoe, Saitama 350-1165, Japan

Tel: +81 49 242 8138

E-mail: s-higurashi@beanstalksnow.co.jp

Online Resource 1. Flowchart of study participant selection

A total of 1,210 mother–infant dyads were initially enrolled in the Japanese Human Milk Study (University Hospital Medical Information Network [UMIN] ID: 000015494). Of these, 1,122 Japanese mother–infant dyads were eligible, and 930 provided sufficient human milk samples at 1–2 months postpartum. From this population, 200 dyads were randomly selected for inclusion in the present analysis. Among the 200 analyzed dyads, incomplete follow-up questionnaire data resulted in missing outcome information for specific conditions during the first year of life: gastroenteritis (*n* = 45), bronchitis (*n* = 47), otitis media (*n* = 45), respiratory syncytial virus infection (*n* = 43), hand, foot, and mouth disease (*n* = 42), exanthem subitum (*n* = 41), food allergy (*n* = 50), and atopic dermatitis (*n* = 48). Dyads with missing information for a given outcome were excluded only from the corresponding disease-specific analyses.

*Abbreviations*: RSV, respiratory syncytial virus; HFMD, hand, foot, and mouth disease; UMIN, University Hospital Medical Information Network.

Online Resource 2. Descriptive characteristics and reported infant outcomes in the overall eligible cohort and dyads whose mothers provided sufficient milk samples^1^

|  | **Overall eligible cohort^2^**  **(N = 1,122)** | | **Provided sufficient milk samples^3^**  **(N = 930)** | |
| --- | --- | --- | --- | --- |
|  | N | n (%) or median (IQR) | N | n (%) or median (IQR) |
| **Infant characteristics** |  |  |  |  |
| **Sex** | 1,122 |  | 930 |  |
| Male |  | 602 (54%) |  | 497 (53%) |
| Female |  | 520 (46%) |  | 433 (47%) |
| **Mode of delivery** | 1,117 |  | 925 |  |
| Vaginal |  | 984 (88%) |  | 819 (89%) |
| Cesarean section |  | 133 (12%) |  | 106 (12%) |
| **Birth weight (g)** | 1,108 | 3,064 (2,839, 3,308) | 917 | 3,062 (2,838, 3,304) |
| **Gestational age (weeks)** | 1,079 |  | 895 |  |
| < 37 |  | 26 (2.4%) |  | 25 (2.8%) |
| 37–41 |  | 1,049 (97%) |  | 866 (97%) |
| ≥ 42 |  | 4 (0.4%) |  | 4 (0.4%) |
| **Feeding status** | 1,117 |  | 927 |  |
| Exclusive breastfeeding (≥ 90%) |  | 963 (86%) |  | 811 (88%) |
| Non-exclusive breastfeeding (< 90%) |  | 154 (14%) |  | 116 (13%) |
| Gastroenteritis | 819 | 141 (17%) | 692 | 117 (17%) |
| Bronchitis | 812 | 84 (10%) | 685 | 70 (10%) |
| Otitis media | 818 | 145 (18%) | 691 | 118 (17%) |
| RSV infection | 825 | 128 (16%) | 700 | 105 (15%) |
| HFMD | 823 | 102 (12%) | 697 | 85 (12%) |
| Exanthem subitem | 837 | 204 (24%) | 704 | 171 (24%) |
| **Allergies** |  |  |  |  |
| Food allergy | 809 | 99 (12%) | 685 | 86 (13%) |
| Atopic dermatitis | 810 | 46 (5.7%) | 686 | 40 (5.8%) |
| **Maternal characteristics** |  |  |  |  |
| **Age at delivery** | 1,122 | 31 (28, 34) | 930 | 31 (28, 34) |
| **Prepregnancy BMI (kg/m^2^)** | 1,111 |  | 922 |  |
| < 18.5 |  | 176 (16%) |  | 147 (16%) |
| 18.5–24.9 |  | 857 (77%) |  | 706 (77%) |
| ≥ 25 |  | 78 (7.0%) |  | 69 (7.5%) |
| **Delivery experience** | 1,117 |  | 925 |  |
| Primiparous |  | 386 (35%) |  | 311 (34%) |
| Multiparous |  | 731 (65%) |  | 614 (66%) |
| **Smoking status** | 1,118 |  | 928 |  |
| Never smoked |  | 778 (70%) |  | 643 (69%) |
| Former smoker (quit before pregnancy) |  | 320 (29%) |  | 268 (29%) |
| Smoked during pregnancy |  | 20 (1.8%) |  | 17 (1.8%) |
| **Education level** | 1,113 |  | 923 |  |
| JHS/HS |  | 246 (22%) |  | 210 (23%) |
| Some college/technical school |  | 469 (42%) |  | 386 (42%) |
| 4-year college or graduate degree |  | 398 (36%) |  | 327 (35%) |
| **Household income from all sources (million JPY/year)** | 1,078 |  | 897 |  |
| < 4 |  | 287 (27%) |  | 241 (27%) |
| 4 to < 8 |  | 637 (59%) |  | 527 (59%) |
| ≥ 8 |  | 154 (14%) |  | 129 (14%) |
| **Parental history of allergic disease^4^** | 1,093 | 746 (68%) | 906 | 615 (68%) |

^1^ Differences between the total N for a variable and the number of dyads reflect missing data for that variable.

^2^The overall eligible cohort consisted of 1,122 mother–infant dyads after exclusion of participants with incomplete or invalid responses and those for whom either the mother or her partner was not Japanese.

^3^ Sufficient milk sample provision was defined as maternal provision of > 50 mL of human milk at 1–2 months postpartum; 930 mother–infant dyads in the overall eligible cohort met this criterion.

^4^ Parental history of allergic disease included asthma, food allergy, atopic dermatitis, allergic rhinitis, allergic conjunctivitis, and urticaria.

Abbreviations: RSV, respiratory syncytial virus; HFMD, hand, foot, and mouth disease; BMI, body mass index; JHS, junior high school; HS, high school; JPY, Japanese yen.

Online Resource 3. Crude Firth logistic regression results between glycan groups in human milk at 1-2 months postpartum and infectious and allergic outcomes during the first year of life

Associations were evaluated using crude Firth logistic regression among the 200 Japanese mother–infant dyads. Explanatory variables included maternal secretor status and glycan groups classified by structural characteristics, including human milk oligosaccharides (HMOs) and N-/O-glycans. Cell colors represent odds ratios (ORs), with red indicating OR > 1 and blue indicating OR < 1; darker shading reflects greater deviation from the null value (OR = 1). Color intensity alone does not indicate statistical significance. Only associations with nominal multiplicity-unadjusted p values < 0.05 are annotated within the cells.

*Abbreviations*: Secretor, maternal secretor status (positive for the functional enzyme encoded by the *FUT2* gene); HMO_total, total human milk oligosaccharides; HMO_Fuc, fucosylated HMO; HighMannose, high-mannose–type N-glycans; Complex, complex-type N-glycans; N_Fuc, fucosylated N-glycans; O_Total, total O-glycans; O_Fuc, fucosylated O-glycans; OR, odds ratio; RSV, respiratory syncytial virus.

Online Resource 4. Associations between glycan groups and infectious and allergic outcomes in adjusted Firth logistic regression models^1^

|  |  | Gastroenteritis | | | |  | Bronchitis | | | |  | Otitis.media | | | | |
| --- | --- | --- | --- | --- | --- | --- | --- | --- | --- | --- | --- | --- | --- | --- | --- | --- |
| Glycans group |  | OR | 95% CI | p value | q value |  | OR | 95% CI | p value | q value |  | OR | 95% CI | p value | q value |  |
| Secretor |  | 1.23 | 0.47-3.73 | 0.69 | 0.77 |  | 0.82 | 0.28-2.85 | 0.73 | 0.92 |  | 1.02 | 0.38-3.12 | 0.97 | 0.97 |  |
| Sialylated glycan |  | 0.85 | 0.52-1.30 | 0.46 | 0.77 |  | 0.46 | 0.20-0.90 | 0.021 | 0.19 |  | 1.22 | 0.79-1.83 | 0.36 | 0.54 |  |
| Total HMO |  | 1.20 | 0.81-1.77 | 0.37 | 0.77 |  | 0.83 | 0.49-1.35 | 0.45 | 0.92 |  | 1.58 | 1.05-2.42 | 0.028 | 0.25 |  |
| Fucosylated HMO |  | 1.29 | 0.86-1.98 | 0.23 | 0.68 |  | 0.90 | 0.57-1.45 | 0.67 | 0.92 |  | 1.50 | 0.97-2.39 | 0.07 | 0.30 |  |
| High-mannose–type N-glycan |  | 0.97 | 0.45-1.46 | 0.92 | 0.92 |  | 1.09 | 0.50-1.65 | 0.72 | 0.92 |  | 0.98 | 0.64-1.33 | 0.90 | 0.97 |  |
| Complex-type N-glycan |  | 0.62 | 0.25-1.10 | 0.12 | 0.53 |  | 0.43 | 0.10-1.08 | 0.09 | 0.39 |  | 1.20 | 0.84-1.67 | 0.29 | 0.54 |  |
| Fucosylated N-glycan |  | 0.51 | 0.19-1.05 | 0.08 | 0.53 |  | 0.78 | 0.29-1.37 | 0.47 | 0.92 |  | 1.07 | 0.73-1.50 | 0.70 | 0.91 |  |
| Total O-glycan |  | 0.90 | 0.43-1.30 | 0.63 | 0.77 |  | 0.94 | 0.32-1.39 | 0.82 | 0.92 |  | 1.16 | 0.83-1.55 | 0.35 | 0.54 |  |
| Fucosylated O-glycan |  | 0.86 | 0.48-1.38 | 0.55 | 0.77 |  | 1.00 | 0.52-1.70 | 0.99 | 0.99 |  | 1.19 | 0.82-1.71 | 0.35 | 0.54 |  |

Online Resource 4 (Continued)

|  |  | RSV infections | | | |  | HFMD | | | |  | Exanthem subitum | | | |
| --- | --- | --- | --- | --- | --- | --- | --- | --- | --- | --- | --- | --- | --- | --- | --- |
| Glycans group |  | OR | 95% CI | p value | q value |  | OR | 95% CI | p value | q value |  | OR | 95% CI | p value | q value |
| Secretor |  | 1.27 | 0.44-4.37 | 0.67 | 0.80 |  | 1.86 | 0.62-7.38 | 0.28 | 0.38 |  | 1.02 | 0.43-2.59 | 0.97 | 0.97 |
| Sialylated glycan |  | 1.03 | 0.64-1.59 | 0.88 | 0.88 |  | 0.77 | 0.45-1.24 | 0.30 | 0.38 |  | 1.26 | 0.87-1.83 | 0.22 | 0.97 |
| Total HMO |  | 1.31 | 0.86-2.00 | 0.20 | 0.80 |  | 0.77 | 0.48-1.19 | 0.24 | 0.38 |  | 1.13 | 0.79-1.61 | 0.51 | 0.97 |
| Fucosylated HMO |  | 1.48 | 0.94-2.42 | 0.09 | 0.80 |  | 0.85 | 0.57-1.30 | 0.45 | 0.51 |  | 1.15 | 0.80-1.66 | 0.46 | 0.97 |
| High-mannose–type N-glycan |  | 1.10 | 0.76-1.51 | 0.57 | 0.80 |  | 0.92 | 0.46-1.27 | 0.63 | 0.63 |  | 0.91 | 0.58-1.23 | 0.54 | 0.97 |
| Complex-type N-glycan |  | 1.16 | 0.77-1.64 | 0.45 | 0.80 |  | 0.52 | 0.18-1.00 | 0.05 | 0.38 |  | 0.96 | 0.65-1.33 | 0.82 | 0.97 |
| Fucosylated N-glycan |  | 1.08 | 0.70-1.55 | 0.68 | 0.80 |  | 0.59 | 0.21-1.09 | 0.12 | 0.38 |  | 0.92 | 0.61-1.28 | 0.64 | 0.97 |
| Total O-glycan |  | 1.10 | 0.74-1.52 | 0.58 | 0.80 |  | 0.67 | 0.25-1.20 | 0.25 | 0.38 |  | 0.97 | 0.62-1.32 | 0.85 | 0.97 |
| Fucosylated O-glycan |  | 1.10 | 0.65-1.73 | 0.71 | 0.80 |  | 0.66 | 0.32-1.15 | 0.15 | 0.38 |  | 1.03 | 0.67-1.53 | 0.90 | 0.97 |

Online Resource 4 (Continued)

|  |  | Food allergy | | | |  | Atopic dermatitis | | | |
| --- | --- | --- | --- | --- | --- | --- | --- | --- | --- | --- |
| Glycans group |  | OR | 95% CI | p value | q value |  | OR | 95% CI | p value | q value |
| Secretor |  | 11.51 | 1.46-1488.11 | 0.014 | 0.13 |  | 0.49 | 0.13-2.11 | 0.31 | 0.56 |
| Sialylated glycan |  | 0.75 | 0.39-1.30 | 0.33 | 0.81 |  | 0.82 | 0.36-1.59 | 0.59 | 0.66 |
| Total HMO |  | 1.14 | 0.68-1.85 | 0.60 | 0.90 |  | 0.74 | 0.35-1.46 | 0.40 | 0.60 |
| Fucosylated HMO |  | 1.27 | 0.76-2.17 | 0.36 | 0.81 |  | 0.66 | 0.36-1.27 | 0.21 | 0.47 |
| High-mannose–type N-glycan |  | 1.04 | 0.56-1.47 | 0.83 | 0.93 |  | 1.51 | 1.06-2.29 | 0.023 | 0.13 |
| Complex-type N-glycan |  | 1.05 | 0.63-1.54 | 0.81 | 0.93 |  | 1.18 | 0.67-1.77 | 0.50 | 0.64 |
| Fucosylated N-glycan |  | 0.83 | 0.31-1.32 | 0.52 | 0.90 |  | 1.38 | 0.90-2.07 | 0.12 | 0.37 |
| Total O-glycan |  | 0.47 | 0.08-1.26 | 0.32 | 0.81 |  | 0.99 | 0.24-1.51 | 0.97 | 0.97 |
| Fucosylated O-glycan |  | 1.03 | 0.53-1.79 | 0.93 | 0.93 |  | 0.25 | 0.04-0.90 | 0.030 | 0.13 |

^1^ Odds ratios (ORs) and 95% confidence intervals (CIs), and multiplicity-unadjusted p values were estimated using Firth logistic regression models adjusted for delivery experience, mode of delivery, infant sex, and birth weight for infection outcomes; parental history of allergic disease was additionally included for allergic outcomes. The q values were calculated within each outcome using the Benjamini–Hochberg false discovery rate procedure to aid interpretation of multiple comparisons.

Abbreviations: OR, odds ratio; CI, confidence interval; Secretor, maternal secretor status (positive for the functional enzyme encoded by the *FUT2* gene); RSV, respiratory syncytial virus; HFMD, hand, foot, and mouth disease.

Online Resource 5. Adjusted Firth logistic regression results for individual glycans carried forward to step 2 of the hierarchical analysis^1^

| Bronchitis | | | | |
| --- | --- | --- | --- | --- |
| Glycans | OR | 95% CI | p value | q value |
| O19 | 0.31 | 0.03-1.16 | 0.12 | 0.25 |
| O24 | 0.62 | 0.10-1.33 | 0.41 | 0.45 |
| O25 | 0.99 | 0.39-1.37 | 0.96 | 0.96 |
| O27 | 0.33 | 0.03-1.23 | 0.17 | 0.29 |
| O28 | 0.77 | 0.29-1.35 | 0.43 | 0.47 |
| N36 | 0.70 | 0.19-1.26 | 0.32 | 0.43 |
| N42 | 0.54 | 0.17-1.14 | 0.13 | 0.25 |
| N51 | 0.22 | 0.03-0.88 | 0.02 | 0.12 |
| N57 | 0.31 | 0.06-0.93 | 0.03 | 0.12 |
| N58 | 0.61 | 0.14-1.39 | 0.30 | 0.43 |
| N66 | 0.52 | 0.14-1.12 | 0.12 | 0.25 |
| 3'-SL | 0.80 | 0.44-1.31 | 0.41 | 0.45 |
| 6'-SL | 0.48 | 0.20-0.92 | 0.02 | 0.12 |
| Otitis media | | | | |
| Glycans | OR | 95% CI | p value | q value |
| 2'-FL | 1.00 | 0.65-1.54 | 0.98 | 0.98 |
| 3-FL | 1.18 | 0.78-1.75 | 0.42 | 0.58 |
| 3'-SL | 1.21 | 0.80-1.78 | 0.35 | 0.56 |
| 6'-SL | 1.35 | 0.92-1.93 | 0.12 | 0.36 |
| LNT | 1.33 | 0.90-1.96 | 0.15 | 0.36 |
| LNnT | 1.21 | 0.82-1.73 | 0.32 | 0.56 |
| LDFT | 1.01 | 0.62-1.57 | 0.96 | 0.98 |
| LNFP-I | 1.30 | 0.89-1.85 | 0.16 | 0.36 |
| LNFP-II | 1.30 | 0.92-1.83 | 0.13 | 0.36 |
| LNDFH-I | 1.02 | 0.66-1.54 | 0.92 | 0.98 |
| LNDFH-II | 1.28 | 0.91-1.77 | 0.15 | 0.36 |
| Atopic dermatitis | | | | |
| Glycans | OR | 95% CI | p value | q value |
| N03 | 1.44 | 0.99-2.07 | 0.06 | 0.15 |
| N12 | 1.50 | 1.02-2.36 | 0.04 | 0.14 |
| N22 | 1.12 | 0.57-1.79 | 0.70 | 0.83 |
| N33 | 1.56 | 1.08-2.39 | 0.02 | 0.14 |
| O01 | 0.39 | 0.09-0.98 | 0.04 | 0.14 |
| O08 | 0.47 | 0.04-1.44 | 0.35 | 0.71 |
| O20 | 0.75 | 0.25-1.42 | 0.42 | 0.71 |
| O24 | 1.00 | 0.21-1.47 | 0.99 | 0.99 |
| O26 | 0.87 | 0.21-1.74 | 0.74 | 0.83 |
| O28 | 0.79 | 0.21-1.49 | 0.56 | 0.80 |
| Food allergy | | | | |
| Glycans | OR | 95% CI | p value | q value |
| LNDFH-II | 0.73 | 0.26-1.27 | 0.33 | 0.44 |
| LNFP-II | 0.30 | 0.07-0.83 | 0.01 | 0.07 |
| LNT | 0.71 | 0.38-1.19 | 0.20 | 0.35 |
| 3-FL | 0.43 | 0.17-0.85 | 0.01 | 0.07 |
| O27 | 0.28 | 0.03-1.21 | 0.16 | 0.35 |
| O26 | 0.34 | 0.04-1.15 | 0.12 | 0.34 |
| O24 | 0.06 | 0.003-0.58 | 0.008 | 0.07 |
| O09 | 0.61 | 0.22-1.25 | 0.20 | 0.35 |
| O08 | 0.33 | 0.05-1.01 | 0.054 | 0.22 |
| N37 | 0.73 | 0.32-1.27 | 0.32 | 0.44 |
| N31 | 0.80 | 0.35-1.33 | 0.44 | 0.51 |
| LNDFH-I | 1.24 | 0.77-1.98 | 0.37 | 0.44 |
| LNFP-I | 1.49 | 0.99-2.24 | 0.06 | 0.22 |
| LDFT | 1.35 | 0.82-2.20 | 0.23 | 0.38 |
| LNnT | 1.49 | 0.94-2.32 | 0.09 | 0.29 |
| 2'-FL | 2.22 | 1.25-4.40 | 0.006 | 0.07 |
| O28 | 0.70 | 0.23-1.28 | 0.31 | 0.44 |
| O20 | 1.39 | 0.87-2.15 | 0.16 | 0.35 |
| O01 | 1.41 | 0.85-2.33 | 0.18 | 0.35 |
| N58 | 1.30 | 0.71-2.18 | 0.36 | 0.44 |
| N42 | 1.06 | 0.63-1.58 | 0.78 | 0.86 |
| N28 | 0.99 | 0.51-1.39 | 0.95 | 0.95 |
| N12 | 1.02 | 0.48-1.44 | 0.91 | 0.95 |

^1^ Odds ratios (ORs) and 95% confidence intervals were estimated using adjusted Firth logistic regression models. For infectious outcomes, adjusted models included delivery experience, mode of delivery, infant sex, and birth weight; for allergic outcomes, parental history of allergic disease was additionally included. Glycan variables were standardized before analysis, and ORs represent associations per one-standard-deviation increase. The p values are unadjusted. The q values were calculated within each outcome using the Benjamini–Hochberg false discovery rate procedure.

Abbreviations: OR, odds ratio; CI, confidence interval; 2’-FL, 2’-fucosyllactose; 3-FL, 3-fucosyllactose; 3’-SL, 3’-sialyllactose; 6’-SL, 6’-sialyllactose; LNT, lacto-*N*-tetraose; LNnT, lacto-*N*-neotetraose; LDFT, lactodifucotetraose; LNFP, lacto-*N*-fucopentaose; LNDFH, lacto-*N*-difucohexaose.

Online Resource 6. Outlier sensitivity analyses for glycan–outcome pairs showing nominal associations in step 2 of the hierarchical analysis^1^

| Outcome | Glycan | Outlier-handling  approach^2^ | OR | 95%CI | p value^3^ |
| --- | --- | --- | --- | --- | --- |
| Bronchitis | N51 | SD3 | 0.22 | 0.03-0.86 | 0.02 |
|  |  | Winsor | 0.23 | 0.03-0.88 | 0.02 |
|  | N57 | SD3 | 0.30 | 0.06-0.96 | 0.04 |
|  |  | Winsor | 0.31 | 0.06-0.93 | 0.03 |
|  | 6’-SL | SD3 | 0.48 | 0.20-0.95 | 0.03 |
|  |  | Winsor | 0.48 | 0.20-0.93 | 0.03 |
| Atopic dermatitis | N12 | SD3 | 1.42 | 0.17-5.24 | 0.67 |
|  |  | Winsor | 1.86 | 0.94-3.55 | 0.07 |
|  | N33 | SD3 | 2.36 | 0.95-5.87 | 0.06 |
|  |  | Winsor | 1.69 | 1.06-2.70 | 0.03 |
|  | O01 | SD3 | 0.39 | 0.09-0.98 | 0.04 |
|  |  | Winsor | 0.39 | 0.09-0.98 | 0.04 |
| Food allergy | LNFP-II | SD3 | 0.30 | 0.07-0.83 | 0.01 |
|  |  | Winsor | 0.30 | 0.07-0.82 | 0.01 |
|  | O24 | SD3 | 0.06 | 0.003-0.56 | 0.01 |
|  |  | Winsor | 0.05 | 0.002-0.57 | 0.01 |
|  | 2’-FL | SD3 | 2.22 | 1.25-4.40 | 0.01 |
|  |  | Winsor | 2.28 | 1.26-4.60 | 0.01 |
|  | 3-FL | SD3 | 0.43 | 0.17-0.88 | 0.02 |
|  |  | Winsor | 0.43 | 0.17-0.85 | 0.01 |

^1^ Sensitivity analyses were conducted for individual glycan–outcome pairs that showed nominal associations in step 2 of the hierarchical analysis. Glycan variables were first standardized using z-score transformation, and potential outliers were then handled based on these standardized values. Odds ratios and 95% confidence intervals were then estimated using Firth logistic regression models with the same outcome-specific covariate adjustments used in the primary analysis. For infection outcomes, adjusted models included delivery experience, mode of delivery, infant sex, and birth weight as covariates. For allergic outcomes, parental history of allergic disease was additionally included.

^2^ Two outlier-handling approaches were applied: exclusion of values beyond 3 standard deviations from the mean and 1% winsorization.

^3^ p values are unadjusted.

Abbreviations: OR, odds ratio; CI, confidence interval; SD3, exclusion of values beyond 3 standard deviations from the mean; Winsor, 1% winsorization; 2’-FL, 2′-fucosyllactose; 3-FL, 3-fucosyllactose; LNFP, lacto-*N*-fucopentaose; 6’-SL, 6’-sialyllactose

Online Resource 7. Reduced-covariate Firth logistic regression sensitivity analyses for individual glycans showing nominal associations in step 2 of the hierarchical analysis^1^

| Outcome | Glycan | Covariates^2^ | OR | 95% CI | p value^3^ |
| --- | --- | --- | --- | --- | --- |
| Bronchitis | 6’-SL | Infant sex | 0.44 | 0.19-0.86 | 0.013 |
|  |  | Delivery method | 0.45 | 0.19-0.88 | 0.016 |
|  |  | Delivery experience | 0.45 | 0.19-0.87 | 0.014 |
|  |  | Birth body weight | 0.46 | 0.19-0.89 | 0.019 |
|  |  | Infant sex, Delivery method | 0.45 | 0.19-0.88 | 0.016 |
|  | N51 | Infant sex | 0.26 | 0.04-0.87 | 0.019 |
|  |  | Delivery method | 0.25 | 0.04-0.91 | 0.026 |
|  |  | Delivery experience | 0.25 | 0.04-0.86 | 0.017 |
|  |  | Birth body weight | 0.24 | 0.04-0.89 | 0.022 |
|  |  | Infant sex, Delivery method | 0.26 | 0.04-0.91 | 0.027 |
|  | N57 | Infant sex | 0.29 | 0.06-0.88 | 0.021 |
|  |  | Delivery method | 0.30 | 0.06-0.92 | 0.028 |
|  |  | Delivery experience | 0.29 | 0.06-0.87 | 0.019 |
|  |  | Birth body weight | 0.28 | 0.06-0.88 | 0.021 |
|  |  | Infant sex, Delivery method | 0.31 | 0.07-0.94 | 0.032 |
| Atopic dermatitis | N12 | Infant sex | 1.51 | 1.09-2.28 | 0.017 |
|  |  | Delivery method | 1.39 | 0.98-2.08 | 0.066 |
|  |  | Delivery experience | 1.41 | 1.03-2.15 | 0.035 |
|  |  | Birth body weight | 1.44 | 1.05-2.05 | 0.028 |
|  |  | Parental history of allergic disease | 1.45 | 1.05-2.15 | 0.027 |
|  |  | Infant sex, Delivery method | 1.47 | 1.01-2.27 | 0.042 |
|  | N33 | Infant sex | 1.60 | 1.15-2.42 | 0.008 |
|  |  | Delivery method | 1.56 | 1.07-2.42 | 0.020 |
|  |  | Delivery experience | 1.53 | 1.09-2.35 | 0.016 |
|  |  | Birth body weight | 1.62 | 1.14-2.44 | 0.009 |
|  |  | Parental history of allergic disease | 1.55 | 1.11-2.32 | 0.012 |
|  |  | Infant sex, Delivery method | 1.57 | 1.09-2.41 | 0.016 |
|  | O01 | Infant sex | 0.43 | 0.11-1.04 | 0.062 |
|  |  | Delivery method | 0.37 | 0.07-0.96 | 0.039 |
|  |  | Delivery experience | 0.38 | 0.10-0.95 | 0.037 |
|  |  | Birth body weight | 0.43 | 0.12-1.03 | 0.059 |
|  |  | Parental history of allergic disease | 0.41 | 0.10-1.01 | 0.054 |
|  |  | Infant sex, Delivery method | 0.39 | 0.08-0.98 | 0.044 |
| Food allergy | 2’-FL | Infant sex | 2.01 | 1.17-3.72 | 0.011 |
|  |  | Delivery method | 2.06 | 1.18-3.92 | 0.009 |
|  |  | Delivery experience | 1.97 | 1.15-3.63 | 0.012 |
|  |  | Birth body weight | 2.11 | 1.20-4.05 | 0.009 |
|  |  | Parental history of allergic disease | 1.99 | 1.16-3.65 | 0.011 |
|  |  | Infant sex, Delivery method | 2.07 | 1.19-3.93 | 0.009 |
|  | 3-FL | Infant sex | 0.46 | 0.19-0.89 | 0.018 |
|  |  | Delivery method | 0.48 | 0.21-0.91 | 0.023 |
|  |  | Delivery experience | 0.48 | 0.21-0.90 | 0.021 |
|  |  | Birth body weight | 0.48 | 0.20-0.91 | 0.021 |
|  |  | Parental history of allergic disease | 0.44 | 0.19-0.87 | 0.014 |
|  |  | Infant sex, Delivery method | 0.46 | 0.19-0.90 | 0.021 |
|  | LNFP-II | Infant sex | 0.39 | 0.11-0.95 | 0.034 |
|  |  | Delivery method | 0.35 | 0.09-0.91 | 0.025 |
|  |  | Delivery experience | 0.40 | 0.11-0.95 | 0.035 |
|  |  | Birth body weight | 0.38 | 0.10-0.93 | 0.028 |
|  |  | Parental history of allergic disease | 0.38 | 0.11-0.95 | 0.034 |
|  |  | Infant sex, Delivery method | 0.34 | 0.09-0.90 | 0.024 |
|  | O24 | Infant sex | 0.09 | 0.01-0.64 | 0.009 |
|  |  | Delivery method | 0.08 | 0.004-0.62 | 0.009 |
|  |  | Delivery experience | 0.12 | 0.01-0.75 | 0.016 |
|  |  | Birth body weight | 0.12 | 0.01-0.84 | 0.026 |
|  |  | Parental history of allergic disease | 0.11 | 0.01-0.71 | 0.014 |
|  |  | Infant sex, Delivery method | 0.06 | 0.003-0.55 | 0.006 |

^1^Sensitivity analyses were conducted for individual glycans that showed nominal associations with each outcome in step 2 of the hierarchical analysis. Odds ratios and 95% confidence intervals were estimated using Firth logistic regression models.

^2^ Models included either one covariate at a time or two covariates, infant sex and mode of delivery, simultaneously. The single covariates examined were infant sex, mode of delivery, delivery experience, and birth weight.

^3^ The p values are nominal p values before false discovery rate correction.

Online Resource 8. Comparison of characteristics of 200 mother–infant dyads according to the maternal secretor status

|  |  | **Total (n = 200)** | **Non-secretor^1^  (n = 39)** | **Secretor^1^  (n = 161)** | **p value^2^** |
| --- | --- | --- | --- | --- | --- |
| **Infant characteristics** | |  |  |  |  |
|  | **Sex** | 200 |  |  | 0.882 |
|  | Male |  | 24 (62%) | 97 (60%) |  |
|  | Female |  | 15 (38%) | 64 (40%) |  |
|  | **Mode of delivery** | 196 |  |  | 0.740 |
|  | Vaginal |  | 37 (95%) | 145 (92%) |  |
|  | Cesarean section |  | 2 (5.1%) | 12 (7.6%) |  |
|  | **Birth weight (g)** | 197 | 3,174  (2,835, 3,329) | 3,071  (2,873, 3,276) | 0.414 |
|  | **Gestational age (weeks)** | 190 |  |  | 0.668 |
|  | < 37 |  | 0 (0%) | 4 (2.6%) |  |
|  | 37–41 |  | 38 (100%) | 147 (97%) |  |
|  | 42 ≤ |  | 0 (0%) | 1 (0.7%) |  |
|  | **Feeding status** | 199 |  |  | 0.437 |
|  | Exclusive breastfeeding (≥ 90%) |  | 32 (82%) | 139 (87%) |  |
|  | Non-exclusive breastfeeding (< 90%) |  | 7 (18%) | 21 (13%) |  |
|  | **Infections** |  |  |  |  |
|  | Gastroenteritis | 155 | 5 (16%) | 25 (20%) | 0.611 |
|  | Bronchitis | 153 | 4 (13%) | 14 (11%) | 0.762 |
|  | Otitis media | 155 | 5 (16%) | 21 (17%) | 0.845 |
|  | RSV infection | 157 | 4 (13%) | 19 (15%) | >0.999 |
|  | HFMD | 158 | 3 (9.4%) | 23 (18%) | 0.226 |
|  | Exanthem subitum | 159 | 8 (25%) | 33 (26%) | 0.909 |
|  | **Allergy** |  |  |  |  |
|  | Food allergy | 150 | 0 (0%) | 18 (15%) | 0.024 |
|  | Atopic dermatitis | 152 | 3 (9.7%) | 7 (5.8%) | 0.427 |
| **Maternal characteristics** | | | | | |
|  | **Age of delivery** | 200 | 32 (28, 35) | 31 (28, 34) | 0.598 |
|  | **Prepregnancy BMI (kg/m^2^)** | 196 |  |  | >0.999 |
|  | < 18.5 |  | 5 (13%) | 23 (15%) |  |
|  | 18.5–24.9 |  | 30 (79%) | 122 (77%) |  |
|  | > 25 |  | 3 (7.9%) | 13 (8.2%) |  |
|  | **Delivery experience** | 198 |  |  | 0.882 |
|  | Primiparous |  | 13 (33%) | 55 (35%) |  |
|  | Multiparous |  | 26 (67%) | 104 (65%) |  |
|  | **Smoking status** | 200 |  |  | 0.711 |
|  | Never smoked |  | 26 (67%) | 109 (68%) |  |
|  | Former smoker (quit before pregnancy) |  | 12 (31%) | 50 (31%) |  |
|  | Smoked during pregnancy |  | 1 (2.6%) | 2 (1.2%) |  |
|  | **Education level (mother)** | 199 |  |  | 0.745 |
|  | JHS/HS |  | 8 (21%) | 42 (26%) |  |
|  | Some college/technical school |  | 17 (44%) | 67 (42%) |  |
|  | 4-year college or graduate degree |  | 14 (36%) | 51 (32%) |  |
|  | **Household income from all sources**  **(million JPY/year)** | 194 |  |  | 0.275 |
|  | < 4 |  | 11 (29%) | 46 (29%) |  |
|  | 4 to < 8 |  | 20 (53%) | 95 (61%) |  |
|  | 8 ≤ |  | 7 (18%) | 15 (9.6%) |  |
|  | **Parental history of allergic diseases (either parent)** |  |  |  |  |
|  | Asthma | 195 | 5 (13%) | 33 (21%) | 0.272 |
|  | Food allergies | 195 | 7 (18%) | 24 (15%) | 0.635 |
|  | Atopic dermatitis | 195 | 7 (18%) | 37 (24%) | 0.496 |
|  | Allergic rhinitis | 195 | 16 (42%) | 92 (59%) | 0.066 |
|  | Allergic conjunctivitis | 195 | 4 (11%) | 19 (12%) | >0.999 |
|  | Urticaria | 195 | 7 (18%) | 18 (11%) | 0.280 |
|  | Any allergic disease | 195 | 25 (66%) | 114 (73%) | 0.404 |

^1^ Values are presented as median (IQR) or number (%).

^2^ p values were calculated by the Wilcoxon rank-sum test for continuous variables and Pearson’s chi-squared test or Fisher’s exact test for categorical variables, as appropriate.

Abbreviations: RSV, respiratory syncytial virus; HFMD, hand, foot and mouth disease; BMI, body mass index; JHS, junior high school; HS, high school; JPY, Japanese yen.

Online Resource 9. Sensitivity analysis of associations between selected individual glycans and food allergy among secretor mothers^1^

| Glycans | OR | 95%CI | p value | q value |
| --- | --- | --- | --- | --- |
| LNFP-II | 0.33 | 0.06-1.26 | 0.11 | 0.18 |
| 3-FL | 0.54 | 0.20-1.28 | 0.17 | 0.18 |
| 2’-FL | 1.68 | 0.79-3.71 | 0.18 | 0.18 |
| O24 | 0.11 | 0.01-0.93 | 0.04 | 0.16 |

^1^Analyses were restricted to secretor mothers. Candidate glycans were selected from individual glycans that showed nominal associations with food allergy in the overall step 2 analysis. Odds ratios and 95% confidence intervals were estimated using adjusted Firth logistic regression models included infant sex, mode of delivery, delivery experience, birth weight, and parental history of allergic disease. Glycan variables were standardized before analysis, and odds ratios represent associations per one-standard-deviation increase in each glycan variable. The p values are unadjusted. The q values were calculated within the food allergy analysis using the Benjamini–Hochberg false discovery rate procedure to aid interpretation of multiple comparisons.

*Abbreviations*: OR, odds ratio; CI, confidence interval; 2′-FL, 2′-fucosyllactose; 3-FL, 3-fucosyllactose; LNT, lacto-*N*-tetrose; LNnT, lacto-*N*-neotetraose; LDFT, lactodifucotetraose; LNFP-I, lacto-*N*-fucopentaose I; LNFP-II, lacto-*N*-fucopentaose II; LNDFH-I, lacto-*N*-difucohexaose I; LNDFH-II, lacto-*N*-difucohexaose II
